# Reconstructing Group Wavelet Transform From Feature Maps With a Reproducing Kernel Iteration

**DOI:** 10.3389/fncom.2022.775241

**Published:** 2022-03-15

**Authors:** Davide Barbieri

**Affiliations:** Departamento de Matemáticas, Universidad Autónoma de Madrid, Madrid, Spain

**Keywords:** harmonic analysis, wavelet analysis, reproducing kernel Hilbert spaces, primary visual cortex, receptive fields, orientation preference maps

## Abstract

In this article, we consider the problem of reconstructing an image that is downsampled in the space of its *SE*(2) wavelet transform, which is motivated by classical models of simple cell receptive fields and feature preference maps in the primary visual cortex. We prove that, whenever the problem is solvable, the reconstruction can be obtained by an elementary project and replace iterative scheme based on the reproducing kernel arising from the group structure, and show numerical results on real images.

## 1. Introduction

This article introduces an iterative scheme for solving a problem of image reconstruction, motivated by the classical behavior of the primary visual cortex (V1), in the setting of group wavelet analysis. The mathematical formulation of the problem is that of the reconstruction of a function on the plane which, once represented as a function on the group *SE*(2) = ℝ^2^ ⋊ 𝕊^1^ of rotations and translations of the Euclidean plane *via* the group wavelet transform, is known only on a certain two-dimensional subset of this three-dimensional group. This problem is equivalent to that of filling in missing information related to a large subset of the *SE*(2) group and ultimately inquires about the completeness of the image representation provided by feature maps observed in V1.

One of the main motivations for the present study comes indeed from neuroscience and the modeling of classical receptive fields of simple cells in terms of group actions restricted to feature maps such as the orientation preference maps. The attempts to model mathematically the measured behavior of the brain's primary visual cortex (V1) have led to the introduction of the linear-nonlinear-Poisson (LNP) model (Carandini et al., 2005), which defines what is sometimes referred to as classical behavior. It describes the activity of a neuron in response to a visual stimulus as a Poisson spiking process driven by a linear operation on the visual stimulus, modeled by the receptive field of the neuron, passed through a sigmoidal nonlinearity. A series of thorough studies of single cell behavior could find a rather accurate description of the receptive fields of a large amount of V1 cells, called simple cells, within the LNP model, in terms of integrals over Gabor functions located in a given position of the visual field (Marcelja, 1980; Ringach, 2002). This description formally reduces the variability in the classical behavior of these cells to few parameters, regulating the position on the visual field, the size, the shape, and the local orientation of a two-dimensional modulated Gaussian. A slightly simplified description of a receptive field activity *F* in response to a visual stimulus defined by a real function *f* on the plane, representing light intensity, is the following: denoting by $x=\left(\begin{array}{c} x_{1}\\ x_{2} \end{array}\right),y=\left(\begin{array}{c} y_{1}\\ y_{2} \end{array}\right)\in {\mathbb{R}}^{2}$,


(1) $$\begin{array}{l} F=s\sqrt{2\pi }\int_{{\mathbb{R}}^{2}}f\left(y\right)e^{-2\pi ip\left(\left(x_{1}-y_{1}\right)\cos\theta +\left(x_{2}-y_{2}\right)\sin\theta \right)}e^{-2\pi^{2}s^{2}|x-y{|}^{2}}dy \end{array}$$

where the parameters *s, p* ∈ ℝ^+^ define the local (inverse) scale and spatial frequency, the angle θ ∈ [0, 2π) defines the local direction and *x* ∈ ℝ^2^ define the local position of the receptive field in the visual field, while the complex formulation can be formally justified by the prevalence of so-called even and odd cells (Ringach, 2002). We will focus on the sole parameters *x*, θ. This may be considered restrictive (Hubel and Wiesel, 1962; Sarti et al., 2008; Barbieri, 2015), but it is nevertheless interesting since angles represent a relevant local feature detected by V1 (Hubel and Wiesel, 1962) whose identification has given rise to effective geometrical models of perception (Petitot and Tondut, 1999; Paul C. Bressloff, 2002; John Zweck, 2004; Citti and Sarti, 2006, 2015). We will give more details below concerning this restriction. In this case, one can then model the linear part of the classical behavior of simple cells in terms of an object that is well-studied in harmonic analysis: by rephrasing Equation (1) as


(2) $$\begin{array}{l} F=F\left(x,\theta \right) \end{array}$$

for fixed values of *p, s*, we obtain a *SE*(2) group wavelet transform of *f* (refer to Section 2). On the other hand, classical experiments with optical imaging have shown that not all parameters θ ∈ 𝕊^1^ are available for these cortical operations (Blasdel and Salama, 1986; Bonhoeffer and Grinvald, 1991; Weliky et al., 1996; Bosking et al., 1997; White and Fitzpatrick, 2007). Furthermore, with single-cell precision two-photon imaging techniques (Ohki et al., 2006), we can say with a good approximation that in many mammals, a pinwheel-shaped function Θ : ℝ^2^ → 𝕊^1^ determines the available angle at each position. This feature map can be introduced in the Model (1) by saying that the receptive fields in V1 record the data.


(3) $$\begin{array}{l} \left\{F\left(x,\Theta \left(x\right)\right)\;:\;x\in {\mathbb{R}}^{2}\right\}. \end{array}$$

Within this setting, a natural question is, thus, whether this data actually contains all the sufficient information to reconstruct the original image *f*, and how can we reobtain *f* from these data. This is the main problem we aim to address. Note that one can equivalently consider the graph $G_{\Theta }=\left\{\left(x,\theta \right)\in {\mathbb{R}}^{2}\times S^{1}:\theta =\Theta \left(x\right)\right\}$ of the feature map Θ instead of the feature map itself: this would allow us to address the problem as that of the injectivity of the restriction of the function (Equation 2) to the subset $G_{\Theta }$, which is the point of view taken in Section 3.

Before proceeding, we recall that a severe limitation of the purely spatial Model (Equation 1) is that of disregarding temporal behaviors (DeAngelis et al., 1995; Cocci et al., 2012). Moreover, the classical behavior described by the LNP model is well-known to be effective only to a limited extent: several other mechanisms are present that describe a substantial amount of the neural activity in V1, such as Carandini-Heeger normalization (Carandini and Heeger, 2012), so-called non-classical behaviors (Fitzpatrick, 2000; Carandini et al., 2005), and neural connectivity (Angelucci et al., 2002). However, spatial receptive fields and the LNP provide relevant insights on the functioning of V1 (Ringach, 2002). Moreover, the ideas behind the LNP model have been a main source of inspiration in other disciplines, notably for the design of relevant mathematical and computational theories, such as wavelets and convolutional neural networks (Marr, 1980; LeCun et al., 2010). We also point out that the use of groups and invariances to describe the variability of the neural activity has proved to be a solid idea to build effective models (Citti and Sarti, 2006; Anselmi and Poggio, 2014; Petitot, 2017), whose influence on the design of artificial learning architectures is still very strong (Anselmi et al., 2019, 2020; Montobbio et al., 2019; Lafarge et al., 2021).

We would like to mention that, in addition, some other simplifications already at the level of modeling the receptive fields of V1 cells are assumed when considering (Equation 3) as the data collected by V1, i.e., by restricting the parameters of interest to only (*x*, θ) and by considering just one angle θ for each position *x*. First, in Equation (1), one performs operations *x* − *y*, meaning that both *x* and *y* are coordinates on the visual plane. On the other hand, it is known that a nontrivial retinotopic map links the cortical coordinates with the visual field (Tootell et al., 1982). Thus, in order for a cortical map Θ to be compared with actual measurements such as those of Blasdel and Salama (1986); Bonhoeffer and Grinvald (1991); Weliky et al. (1996); Bosking et al. (1997); Ohki et al. (2006); White and Fitzpatrick (2007), in Equation (3), we should not consider Θ as computed directly on the visual field coordinate *x* but rather compose Θ with the retinotopic map. Equation (3) is, thus, to be considered, from the strictly neural point of view, either as a local approximation or as a formulation where Θ also contains the retinotopic map. Moreover, we are assuming yet another simplification of what would be a realistic model by considering the parameters *p, s* in Equation (1) as fixed. More specifically, it is known that the spatial frequency *p* of the receptive field is itself a feature that is organized in a columnar fashion, i.e., by a cortical map (Ribot et al., 2013), which is correlated with that of orientations, and recent refined mathematical models are able to reproduce these cortical maps on the basis of the sole structure of the receptive field (Baspinar et al., 2018). Concerning the (inverse) scale parameter *s*, it is known (Hubel and Wiesel, 1974) that it does not only vary horizontally on the cortex, but it also changes along the transverse direction, hence providing a local analysis at more than one scale. Moreover, considering the variation of the transversal average local scale with respect to the horizontal cortical coordinates, it has been observed that, up to the scatter variations, this scale is also correlated with another cortical map, the retinotopic map (Harvey and Dumoulin, 2011). Finally, the two parameters *p* and *s* appear to be statistically correlated in the populations of simple cells whose receptive fields have been measured and fitted with the Gabor model (Ringach, 2002). If these assumptions are considered satisfactory, however, it becomes easy to reconcile a description such as that of Equation (3) with other classical ones based on the concept of tuning curves (refer to e.g., Webster and Valois, 1985. An orientation tuning curve for a given V1 cell is a measured bell-shaped curve that shows the response of the cell to different orientations. That is, it describes how a cell that has a given orientation preference θ_0_ actually has a nonzero activation when a visual stimulus with similar but not equally oriented stimuli are presented in its receptive field area. In this sense, each cell is not sensitive to just one orientation, in the same way as it is not sensitive to just one point in the visual space. These areas of influence in the parameter space (*x*, θ) for a given cell of V1 can actually be related to the structure of receptive fields and the uncertainty principle of the *SE*(2) group (Barbieri et al., 2014), and orientation tuning curves too can be obtained from Gabor-like receptive fields (Barbieri, 2015).

For the sake of completeness and previously unfamiliar readers, we also recall that simple cells receptive fields are sometimes modeled as scaled directional derivatives of Gaussians. This model, which can actually reproduce quite closely certain Gabor functions, was initially introduced for its theoretical interest and its neural plausibility (Webster and Valois, 1985; Young, 1985; Koenderink and van Doorn, 1987) and later used extensively in the scale space theory in computer vision (Florack et al., 1992; Lindeberg, 1994) as well as for a largely developed axiomatic theory of receptive fields (Lindeberg, 2013, 2021). A thorough discussion on the relationship between this model and that of Gabor functions can be found in (Petitot, 2017, Ch. 3).

Another motivation for studying the completeness of the data collected with Equation (3) comes from the relationship of this problem with that of sampling in reproducing kernel Hilbert spaces (RKHS). The RKHS structure, in this case, is that of the range of the group wavelet transform, and will be discussed in detail, together with the basics on the *SE*(2) wavelet transform, in Section 2. Reducing the number of parameters in a wavelet transform is a common operation, that one typically performs for discretization purposes (refer to e.g., Daubechies, 1992), or e.g., because it is useful to achieve square-integrability (Ali et al., 1991). The main issue is that these operations are typically constrained nontrivially in order to retain sufficient information that allows one to distinguish all interesting signals. For example, when discretizing the short-time Fourier transform to obtain a discrete Gabor Transform, the well-known density theorem (Heil, 2007) imposes a minimum density of points in phase space where the values of the continuous transform must be known in order to retain injectivity for signals of finite energy. Much is known about this class of problems in the context of sampling, from classical Shannon's theorem and uncertainty-related signal recovery results (Donoho and Stark, 1989) to general sampling results in RKHS (Fuhr et al., 2017; Grochenig et al., 2018). However, the kind of restriction implemented by feature maps does not seem to fit into these settings, even if some similarities may be found between the pinwheel-shaped orientation preference maps (Bonhoeffer and Grinvald, 1991) and Meyer's quasicrystals, which have been recently used for extending compressed sensing results (Candes et al., 2006; Matei and Meyer, 2010; Agora et al., 2019).

We remark that this article does not focus on dictionary learning techniques for image representation that may give rise to realistic receptive fields and feature maps, such as e.g., those studied in Hyvarinen and Hoyer (2001) and Miikkulainen et al. (2005). We will consider only the problem of reconstruction of the *SE*(2) wavelet transform from a given feature map.

The plan of the article is as follows. In Section 2, after a brief overview of the *SE*(2) transform and its RKHS structure, we will formalize in a precise way the problem of functional reconstruction after the restriction (Equation 3) has been performed. In Section 3, we will introduce a technique to tackle this problem, given by an iterative kernel method based on projecting the restricted *SE*(2) wavelet transform onto the RKHS defined by the group representation. Moreover, we will consider a discretization of the problem and, in the setting of finite dimensional vector spaces, we will give proof that the proposed iteration converges to the desired solution. Finally, in Section 4, we will show and discuss numerical results on real images.

## 2. Overview of the *SE*(2) Transform

The purpose of this section is to review the fundamental notions of harmonic analysis needed to provide a formal statement of the problem. We will focus on the group wavelet transform defined by the action on *L*^2^(ℝ^2^) of the group of rotations and translation of the Euclidean plane, expressed as a convolutional integral transform.

We will denote the Fourier transform of *f* ∈ *L*^1^(ℝ^2^) ∩ *L*^2^(ℝ^2^) by


$$\hat{f}\left(\xi \right)=\int_{{\mathbb{R}}^{2}}e^{-2\pi ix.\xi }f\left(x\right)dx$$

and, as customary, we will use the same notation for its extension by density to the whole *L*^2^(ℝ^2^). We will also denote by * the convolution on ℝ^2^:


$$\left(f*g\right)\left(x\right)=\int_{{\mathbb{R}}^{2}}f\left(y\right)g\left(x-y\right)dy.$$

Let 𝕊^1^ be the abelian group of angles of the unit circle, which is isomorphic either to the one dimensional torus 𝕋 = [0, 2π) or to the group *SO*(2) of rotations of the plane ℝ^2^. The group *SE*(2) = ℝ^2^ ⋊ 𝕊^1^ is (refer to e.g., Sugiura, 1990, Ch. IV) the semidirect product group with elements (*x*, θ) ∈ ℝ^2^ × 𝕊^1^ and composition law


$$\left(x,\theta \right)\cdot \left(x^{\prime },\theta^{\prime }\right)=\left(x+r_{\theta }x^{\prime },\theta +\theta^{\prime }\right)$$

where $r_{\theta }=\left(\begin{array}{ll} \cos\theta & -\sin\theta \\ \sin\theta & \cos\theta \end{array}\right)$. Its Haar measure, which is the (Radon) measure on the group which is invariant under group operations, is the Lebesgue measure on ℝ^2^ ⋊ 𝕊^1^.

A standard way to perform a wavelet analysis with respect to the *SE*(2) group on two-dimensional signals is given by the operator defined as follows.

**D****EFINITION 1**. *Let us denote by* $R:S^{1}\to U\left(L^{2}\left({\mathbb{R}}^{2}\right)\right)$ *the unitary action by rotations of* 𝕊^1^ *on L*^2^(ℝ^2^):


$$R\left(\theta \right)f\left(x\right)=f\left(r_{\theta }^{-1}x\right),\;f\in L^{2}\left({\mathbb{R}}^{2}\right),\;\theta \in {𝕊}^{1}.$$

*Let ψ ∈ L*^2^(ℝ^2^), *and denote by ψ_θ_ = R*(*θ*)*ψ. The SE*(2) *wavelet transform on L*^2^(ℝ^2^) *with the mother wavelet ψ is*


(4) $$\begin{array}{l} W_{\psi }f\left(x,\theta \right)=\left(f*\psi_{\theta }\right)\left(x\right),\;f\in L^{2}\left({\mathbb{R}}^{2}\right). \end{array}$$

In terms of this definition, we can then see that if we choose *s, p* ∈ ℝ^+^, and let $\psi_{s,p}\in L^{2}\left({\mathbb{R}}^{2}\right)$ be


(5) $$\begin{array}{l} \psi_{s,p}\left(x\right)=s\sqrt{2\pi }e^{-2\pi ipx_{1}}e^{-2\pi^{2}s^{2}|x{|}^{2}}, \end{array}$$

we can write (Equation 1) as *F* = *W*_ψ_*s, p*__*f*(*x*, θ).

Moreover, by making use of the quasiregular representation of the *SE*(2) group


(6) $$\begin{array}{l} \Pi \left(x,\theta \right)f\left(y\right)=f\left(r_{\theta }^{-1}\left(y-x\right)\right),\;f\in L^{2}\left({\mathbb{R}}^{2}\right) \end{array}$$

and denoting by $\psi^{†}\left(x\right)=\bar{\psi \left(-x\right)}$, we can rewrite (Equation 4) as follows:


$$W_{\psi }f\left(x,\theta \right)={\langle f,\Pi \left(x,\theta \right)\psi^{†}\rangle }_{L^{2}\left({\mathbb{R}}^{2}\right)}$$

which is a standard form to write the so-called group wavelet transform (refer to e.g., Führ, 2005; Deitmar and Echterhoff, 2014). Note that, in the interesting case (Equation 5), we have $\psi_{s,p}^{†}=\psi_{s,p}$.

The *SE*(2) transform (Equation 4), together with the notion of group wavelet transform, has been studied in multiple contexts (refer to e.g., Weiss and Wilson, 2001; Antoine et al., 2004; Führ, 2005; Duits et al., 2007; Deitmar and Echterhoff, 2014; Dahlke et al., 2015 and references therein), and several of its properties are well-known. In particular, if *W*_ψ_*f* is a bounded operator from *L*^2^(ℝ^2^) to *L*^2^(ℝ^2^ × 𝕊^1^), which happens e.g., for ψ ∈ *L*^1^(ℝ^2^) ∩ *L*^2^(ℝ^2^) by Young's convolution inequality and the compactness of 𝕊^1^, it is easy to see that its adjoint reads


(7) $$\begin{array}{l} W_{\psi }^{*}F\left(x\right)=\int_{{𝕊}^{1}}\left(F\left(\cdot ,\theta \right)*\psi_{\theta }^{†}\right)\left(x\right)d\theta . \end{array}$$

It is also well-known (Weiss and Wilson, 2001) that *W*_ψ_*f* cannot be injective on the whole *L*^2^(ℝ^2^), i.e., we cannot retrieve an arbitrary element of *L*^2^(ℝ^2^) by knowing its *SE*(2) transform. However, as shown in Duits (2005); Duits et al. (2007), and applied successfully in a large subsequent series of works (e.g., Duits and Franken, 2010; Zhang et al., 2016; Lafarge et al., 2021), it is possible to obtain a bounded invertible transform by extending the notion of *SE*(2) transform to mother wavelets ψ that do not belong to *L*^2^(ℝ^2^), or by simplifying the problem and reduce the wavelet analysis to the space of bandlimited functions, that are those functions whose Fourier transform is supported on a bounded set, whenever a Calderón's admissibility condition holds. Since our main point in this article is not the reconstruction of the whole *L*^2^(ℝ^2^), we will consider the *SE*(2) transform with this second, simplified, approach. In this case, the image of the *SE*(2) transform is a reproducing kernel Hilbert subspace of *L*^2^(ℝ^2^ × 𝕊^1^) whose kernel will play an important role in the next section. For convenience, we formalize these statements with the next two theorems, and provide a sketch of the proof in the Appendix, even if they can be considered standard material.

**T****HEOREM 2**. *For R > 0, let* $B_{R}=\left\{\xi \in {\mathbb{R}}^{2}:|\xi |<R\right\}$ *and let*


(8) $$\begin{array}{l} H_{R}=\left\{f\in L^{2}\left({\mathbb{R}}^{2}\right)\;:\text{ supp}\hat{f}\subset B_{R}\right\}. \end{array}$$

*The *SE*(2) wavelet transform (Equation 4) for a mother wavelet ψ ∈ L*^2^(ℝ^2^) *is a bounded injective operator from* $H_{R}$ *to L*^2^(ℝ^2^ × 𝕊^1^) *if and only if there exist two constants 0 < *A* ≤ *B* < ∞ such that*


(9) $$\begin{array}{l} A\leq \int_{{𝕊}^{1}}|\hat{\psi }\left(r_{\theta }^{-1}\xi \right){|}^{2}d\theta \leq B \end{array}$$

*holds for almost every ξ* ∈ *B*_*R*_.

Before stating the next result, we repeat the observation of Weiss and Wilson (2001) and show, using this theorem, that the *SE*(2) transform cannot be a bounded injective operator on the whole *L*^2^(ℝ^2^). Indeed, using that


$$\begin{array}{l} \hat{\psi_{\theta }}\left(\xi \right)=\int_{{\mathbb{R}}^{2}}e^{-2\pi ix.\xi }\psi \left(r_{\theta }^{-1}x\right)dx\\ \;=\int_{{\mathbb{R}}^{2}}e^{-2\pi ix.\left(r_{\theta }^{-1}\xi \right)}\psi \left(x\right)dx=\hat{\psi }\left(r_{\theta }^{-1}\xi \right). \end{array}$$

we can see that the Calderón's function in condition (Equation 9) is actually a radial function


(10) $$\begin{array}{l} C_{\psi }\left(\xi \right)=\int_{{𝕊}^{1}}|\hat{\psi }\left(r_{\theta }^{-1}\xi \right){|}^{2}d\theta \\ \;=\int_{{𝕊}^{1}}|\hat{\psi }\left(|\xi |\cos\varphi ,|\xi |\sin\varphi \right){|}^{2}d\varphi =C_{\psi }\left(\left|\xi \right|\right) \end{array}$$

which, by Plancherel's theorem, satisfies $\int_{0}^{\infty }C_{\psi }\left(\rho \right)\rho d\rho =||\psi |{|}_{L^{2}\left({\mathbb{R}}^{2}\right)}^{2}.$ Hence, the lower bound in condition (Equation 9) cannot be satisfied on the whole ℝ^2^ by any ψ ∈ *L*^2^(ℝ^2^).

On the other hand, for the mother wavelet (Equation 5), since $\hat{\psi }\left(\xi \right)=\frac{1}{s\sqrt{2\pi }}e^{-|\xi +\left(\begin{array}{c} p\\ 0 \end{array}\right){|}^{2}/2s^{2}}$, we have


$$\begin{array}{l} C_{\psi }\left(\xi \right)=\int_{{𝕊}^{1}}|\hat{\psi_{\theta }}\left(r_{\theta }^{-1}\xi \right){|}^{2}d\theta =\frac{1}{2\pi s^{2}}\int_{{𝕊}^{1}}e^{-|\xi +\left(\begin{array}{c} p\;\cos\;\theta \\ p\;\sin\;\theta \end{array}\right){|}^{2}/s^{2}}d\theta \\ =\frac{e^{-\frac{|\xi {|}^{2}+p^{2}}{s^{2}}}}{2\pi s^{2}}\int_{{𝕊}^{1}}e^{-\frac{|\xi |p}{s^{2}}\cos\alpha }d\alpha . \end{array}$$

From here, we can see that *C*_ψ_(ξ) > 0 for all ξ ∈ ℝ^2^, so, even if *C*_ψ_(ξ) → 0 as |ξ| → ∞, we have that the lower bound in Equation (9) is larger than zero for any finite *R*.

The next theorem shows how to construct the inverse of the *SE*(2) wavelet transform on bandlimited functions, and what is the structure of the closed subspace defined by its image.

**T****HEOREM 3**. *Let ψ* ∈ *L*^2^(ℝ^2^) *and R* > 0 *be such that Equation (9) holds. Let also γ* ∈ *L*^2^(ℝ^2^) *be defined by*


(11) $$\begin{array}{l} \hat{\gamma }\left(\xi \right)=\frac{\chi_{B_{R}}\left(\xi \right)}{C_{\psi }\left(\xi \right)}\hat{\psi }\left(\xi \right) \end{array}$$

*where* $\chi_{B_{R}}\left(\xi \right)=\{\begin{array}{ll} 1 & \xi \in B_{R}\\ 0 & \text{otherwise} \end{array}$, and let $H_{R}$ *be as in Equation (8). Then*

*(i) For all* $f\in H_{R}$, *it holds* $W_{\gamma }^{*}W_{\psi }f=f$.
*(ii) The space* $W_{\psi }\left(H_{R}\right)$ *is a reproducing kernel Hilbert subspace of continuous functions of L*^2^(ℝ^2^ × 𝕊^1^), *and the orthogonal projection* ℙ *of L*^2^(ℝ^2^ × 𝕊^1^) *onto* $W_{\psi }\left(H_{R}\right)$ *is*
  (12) $$\begin{array}{l} \mathbb{P}F\left(x,\theta \right)=W_{\psi }W_{\gamma }^{*}F\left(x,\theta \right)\\ =\int_{{𝕊}^{1}}\left(F\left(\cdot ,\theta^{\prime }\right)*\psi_{\theta }*\gamma_{\theta^{\prime }}^{†}\right)\left(x\right)d\theta^{\prime }\;,\;F\in L^{2}\left({\mathbb{R}}^{2}\times {𝕊}^{1}\right). \end{array}$$

Since $W_{\psi }\left(H_{R}\right)$ is a Hilbert space of continuous functions, it makes sense to consider its values on the zero measure set provided by the graph of a function Θ : ℝ^2^ → 𝕊^1^, as in Equation (3). We can then provide a formal statement of the problem discussed in Section 1:


(13) $$\begin{array}{l} \text{for }f\in H_{R}\text{ and }\Theta \;:\;{\mathbb{R}}^{2}\to {𝕊}^{1}\text{, reconstruct }f\\ \text{using only the values }W_{\psi }f\left(x,\Theta \left(x\right)\right). \end{array}$$

For this problem to be solvable, the graph $G_{\Theta }=\left\{\left(x,\theta \right)\in {\mathbb{R}}^{2}\times S^{1}:\theta =\Theta \left(x\right)\right\}$ must be a set of uniqueness for $W_{\psi }\left(H_{R}\right)$. That is, the following condition must hold:


(14) $$\begin{array}{l} \text{if }F\in W_{\psi }\left(H_{R}\right)\text{ and }F{|}_{G_{\Theta }}=0,\text{then }F=0. \end{array}$$

Indeed, if this was not the case, for any nonzero $F\in W_{\psi }\left(H_{R}\right)$ that vanishes on $G_{\Theta }$, the function $f_{F}=W_{\gamma }^{*}\left(W_{\psi }f+F\right)\in H_{R}$ would be different from *f* but *W*_ψ_*f*_*F*_ would coincide with *W*_ψ_*f* on $G_{\Theta }$. That is, we could not solve the problem (Equation 13).

Condition (Equation 14) is in general hard to be checked, and the formal characterization of the interplay between ψ and Θ that makes it hold true is out of the scope of this article. However, in the next section, we will provide a technique for addressing (Equation 13) in a discrete setting, which will allow us to explore in Section 4, the behavior of this problem for various functions Θ inspired by the feature maps measured in V1.

## 3. A Reconstruction Algorithm

In this section, we describe the discretization of the problem (Equation 13) which is used in the next section. Then, we introduce a kernel based iterative algorithm and prove its convergence to the solution whenever the solvability condition (Equation 14) holds.

### 3.1. Discretization of the Problem

The setting described in Section 2 can be discretized in a standard fashion by replacing *L*^2^(ℝ^2^) with ℂ^*N*×*N*^, endowed with the usual Euclidean scalar product, circular convolution, and discrete Fourier transform (FFT), which amounts to replacing ℝ with ℤ_*N*_, the integers modulo *N*. Explicitly, given *f*, ψ ∈ ℂ^*N*×*N*^, $x=\left(\begin{array}{c} x_{1}\\ x_{2} \end{array}\right),y=\left(\begin{array}{c} y_{1}\\ y_{2} \end{array}\right),\xi =\left(\begin{array}{c} y_{1}\\ \xi_{2} \end{array}\right)\in {\mathbb{Z}}_{N}\times {\mathbb{Z}}_{N}$, we have


$$\begin{array}{l} f*\psi \left(x\right)=\overset{N-1}{\underset{y_{1}=0}{\sum }}\overset{N-1}{\underset{y_{2}=0}{\sum }}f\left(y\right)g\left(\left(x-y\right)\;\mathrm{mod}\;N\right),\text{ and }\hat{f}\left(\xi \right)\\ =\overset{N-1}{\underset{x_{1}=0}{\sum }}\overset{N-1}{\underset{x_{2}=0}{\sum }}e^{-2\pi i\frac{x_{1}\xi_{1}+x_{2}\xi_{2}}{N}}f\left(x\right). \end{array}$$

With a uniform discretization of angles, i.e., by replacing 𝕊^1^ with $\frac{2\pi }{M}{\mathbb{Z}}_{M}=\left\{0,\frac{2\pi }{M},\frac{4\pi }{M},\ldots ,2\pi \frac{M-1}{M}\right\}$, we obtain the following discretization of the *SE*(2) transform with the mother wavelet (Equation 5):


$$W_{\psi }f\left(x,j\right)=f*\psi_{\theta_{j}}\left(x\right),\text{where }\hat{\psi_{\theta_{j}}}\left(\xi \right)=e^{-{\left|\xi +\left(\begin{array}{c} p\;\cos\;\theta_{j}\\ p\;\sin\;\theta_{j} \end{array}\right)\right|}^{2}/2s^{2}}$$

for *x*, ξ ∈ ℤ_*N*_ × ℤ_*N*_ and $\theta_{j}=\frac{2\pi }{M}j$, *j* = 0, …, *M*. Thus, in particular, $W_{\psi }f\in {\mathbb{C}}^{N\times N\times M}$. Note that here, for simplicity, we have removed the normalization used in Equation (5).

This allows us to process *N* × *N* digital images while retaining the results of Theorems 2 and 3 as statements on finite frames (refer to e.g., Casazza et al., 2013) since Plancherel's theorem and Fourier convolution theorem still holds. In particular, when computing numerically the inverse of *W*_ψ_ using (i), Theorem 3, one has to choose an *R* > 0 so that Calderón's condition (Equation 9) for


(15) $$\begin{array}{l} C_{\psi }\left(\xi \right)=\overset{M-1}{\underset{j=0}{\sum }}e^{-{\left|\xi +\left(\begin{array}{c} p\;\cos\;\theta_{j}\\ p\;\sin\;\theta_{j} \end{array}\right)\right|}^{2}/2s^{2}} \end{array}$$

holds with some 0 < *A* ≤ *B* < ∞ for all $\xi \in B_{R}=\left\{\xi \in {\mathbb{Z}}_{N}\times {\mathbb{Z}}_{N}:\xi_{1}^{2}+\xi_{2}^{2}<R^{2}\right\}$. This is the injectivity condition on $H_{R}=\left\{f\in {\mathbb{C}}^{N\times N}:\hat{f}\left(\xi \right)=0\forall \xi \notin B_{R}\right\}$ and, due to the finiteness of the space, now it can be achieved for all *R*, i.e., without bandlimiting. However, since this is equivalent to the frame inequalities (Equation 24), the quantity $\sqrt{\frac{B}{A}}$ defines actually the condition number of *W*_ψ_. Thus, in order to keep stability for the inversion, the ratio $\frac{B}{A}$ cannot be arbitrarily large (refer to e.g., Duits et al., 2007; Casazza et al., 2013). Once the parameters *s, p, R* are chosen in such a way that this ratio provides an acceptable numerical accuracy, one can then compute the projection ℙ given by (ii), Theorem 3, on *F* ∈ ℂ^*N*×*N*×*M*^, by making use of Fourier convolution theorem:


(16) $$\begin{array}{l} \hat{\mathbb{P}F}\left(\xi ,j\right)=\frac{\chi_{B_{R}}\left(\xi \right)}{C_{\psi }\left(\xi \right)}\hat{\psi_{\theta_{j}}}\left(\xi \right)\overset{M-1}{\underset{\ell =0}{\sum }}\hat{F}\left(\xi ,\ell \right)\bar{\hat{\psi_{\theta_{\ell }}}\left(\xi \right)}. \end{array}$$

We note at this point that this standard discretization, in general (for *M* different from 2 or 4), retains all of the approaches of Section 2 but the overall semidirect group structure of ℝ^2^ ⋊ 𝕊^1^.

Let us now consider the discretization of the problem (Equation 13) and denote the graph of Θ : ℤ_*N*_ × ℤ_*N*_ → ℤ_*M*_ by $G_{\Theta }$ = {(*x, j*) ∈ ℤ_*N*_ × ℤ_*N*_ × ℤ_*M*_ : *j* = Θ(*x*)}. If we denote by 𝕆_Θ_ the selection operator that sets to zero all the components of an *F* ∈ ℂ^*N*×*N*×*M*^ that do not belong to $G_{\Theta }$, i.e.,


$$\begin{array}{l} {𝕆}_{\Theta }F\left(x,j\right)=\{\begin{array}{cc} F\left(x,j\right) & \left(x,j\right)\in G_{\Theta }\\ 0 & \left(x,j\right)\notin G_{\Theta } \end{array} \end{array}$$

We can see that this is now an orthogonal projection of ℂ^*N*×*N*×*M*^. Hence, problem (Equation 13) can be rewritten in the present discrete setting as follows: given $f\in H_{R}$, find *F* ∈ ℂ^*N*×*N*×*M*^ that solves the linear problem


(17) $$\begin{array}{l} \{\begin{array}{l} \;\mathbb{P}F=F\\ {𝕆}_{\Theta }F={𝕆}_{\Theta }W_{\psi }f. \end{array} \end{array}$$

The meaning of Equation (17) is indeed to recover *W*_ψ_*f*, and hence *f*, knowing only the values *W*_ψ_*f*(*x*, Θ(*x*)).

We propose to look for such a solution using the following iteration in ℂ^*N*×*N*×*M*^: for *F*_0_ = 𝕆_Θ_ *W*_ψ_*f*, compute


(18) $$\begin{array}{l} F_{n}=\mathbb{P}F_{n-1}-{𝕆}_{\Theta }\mathbb{P}F_{n-1}+F_{0}\;,\;n=1,2,\ldots \end{array}$$

The idea behind this iteration is elementary: we start with the values of *W*_ψ_*f* selected by Θ, we project them on the RKHS defined by the image of *W*_ψ_, and we replace the values on $G_{\Theta }$ of the result with the known values of *W*_ψ_*f*. The convergence of this iteration is discussed in the next section. Before that, we observe that Equation (18) can be seen as a linearized version of the Wilson, Cowan, and Ermentrout equation (Wilson and Cowan, 1972; Ermentrout and Cowan, 1980). Indeed, denoting by *K* = (**1** − 𝕆_Θ_)ℙ, Equation (18) can be seen as a forward Euler scheme (time discretization) for the vector valued ordinary differential equation.


$$\frac{d}{dt}F\left(t\right)=-F\left(t\right)+KF\left(t\right)+F_{0}.$$

Apart from the absence of a sigmoid, this is indeed a classical model of population dynamics. Here, the “kernel” *K* is not just the reproducing kernel ℙ, but it also contains the projection on a feature map 𝕆_Θ_. Returning to the model of V1, here, the forcing term *F*_0_ is the data collected by simple cells, while the stationary solution *F*, if it exists, is the full group representation of the visual stimulus defined as the solution to the Volterra-type equation *F* = *KF* + *F*_0_.

### 3.2. The Project and Replace Iteration

We show here that, whenever the problem (Equation 17) is solvable, the iteration (Equation 18) converges to its solution. Since the argument is general, we will consider in this subsection the setting of an arbitrary finite dimensional vector space *V* endowed with a scalar product and the induced norm, and two arbitrary orthogonal projections *P, Q*. For an orthogonal projection *P*, we will denote by *P*^⊥^ = **1** − *P* the complementary orthogonal projection. We will also denote by Ran the range, or image, and by Ker the kernel, of a matrix.

We start with a basic observation, which is just a restatement of the solvability condition (Equation 14) as that of a linear system defined by an orthogonal projection, in this case, *Q*, on a subspace, in this case, characterized as Ran(*P*). The simple proof is included for convenience, and it can be found in the Appendix.

**L****EMMA 4**. *Let *P, Q* be orthogonal projections of a finite dimensional vector space *V*. The system*


(19) $$\begin{array}{l} \{\begin{array}{l} \;PF=F\\ QF=Q\tilde{F} \end{array} \end{array}$$

*has a unique solution in *V* for any* $\tilde{F}\in \text{Ran}\left(P\right)$ *if and only if*


(20) $$\begin{array}{l} \text{Ker}\left(Q\right)\cap \text{Ran}\left(P\right)=\left\{0\right\}. \end{array}$$

The problem posed by the system (Equation 19) is a problem of linear algebra: if we know that a vector *F* belongs to a given subspace Ran(*P*)⊂*V*, and we know the projection of *F* on a different subspace Ran(𝕆)⊂*V*, can we recover *F*? The next theorem shows that, if the system (Equation 19) has a unique solution, such a solution can be obtained by the project and replace iteration (Equation 18). Its proof is given in the Appendix.

**T****HEOREM 5**. *Let *V* be finite dimensional vector space, and let *P, Q* be orthogonal projections of *V*. Given* $\tilde{F}\in \text{Ran}\left(P\right)$, *set* $F_{0}=Q\tilde{F}$, *and let* {*F*_*n*_}_*n*∈ℕ_, {*H*_*n*_}_*n*∈ℕ_ ⊂ *V be the sequences defined by the project and replace the iteration*.


(21) $$\begin{array}{l} \{\begin{array}{l} H_{n}=PF_{n-1}\\ F_{n}=Q^{⊥}H_{n}+F_{0} \end{array}\;,\;n=1,2,\ldots \end{array}$$

*If condition (Equation 20) holds, then*


$$\underset{n\to \infty }{\lim}H_{n}=\underset{n\to \infty }{\lim}F_{n}=\tilde{F}$$

*and the errors* $\left||\tilde{F}-H_{n}|\right|$, $\left||\tilde{F}-F_{n}|\right|$ *decay exponentially with the number of iterations n*.

## 4. Numerical Results

**Figure 1:**
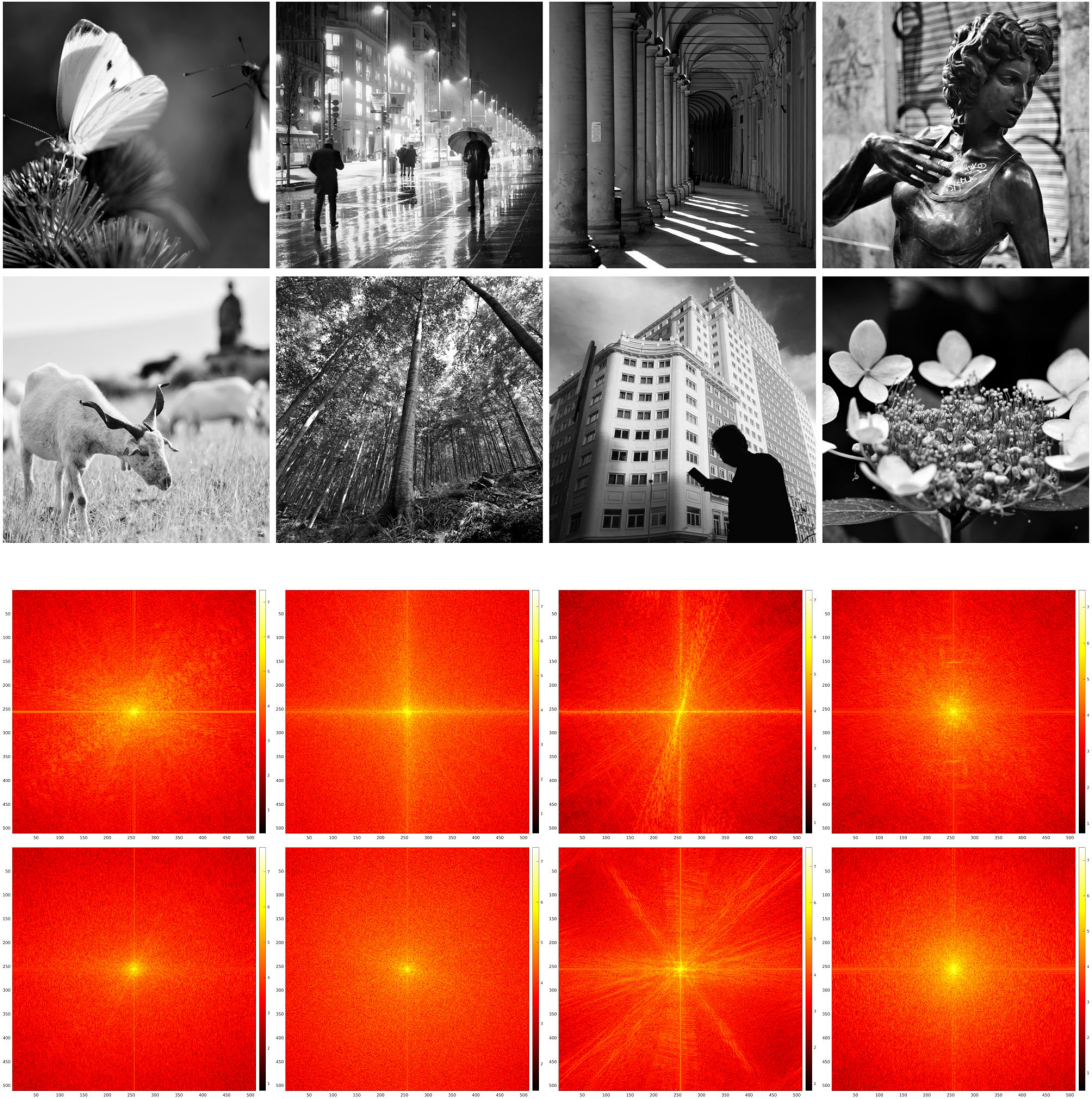
Original test images **(top)** and their Fourier spectra in log_10_ scale **(bottom)**.

We present in this study the reconstruction results of the project and replace iteration on the restriction to feature maps of the *SE*(2) transform of real images. We have chosen eight 512 × 512 pixels, 8-bit grayscale digital images ${\left\{f_{i}\right\}}_{i=1}^{8}\subset {\left\{0,\ldots ,255\right\}}^{512\times 512}$, which are shown in Figure 1 together with their Fourier spectra. Note that, for processing, they have been bandlimited in order to formally maintain the structure described in Section 2. However, this bandlimiting has minimal effects, not visible to the eye, since the spectra have a strong decay: for this reason, the bandlimited images are not shown.

**Figure 2:**
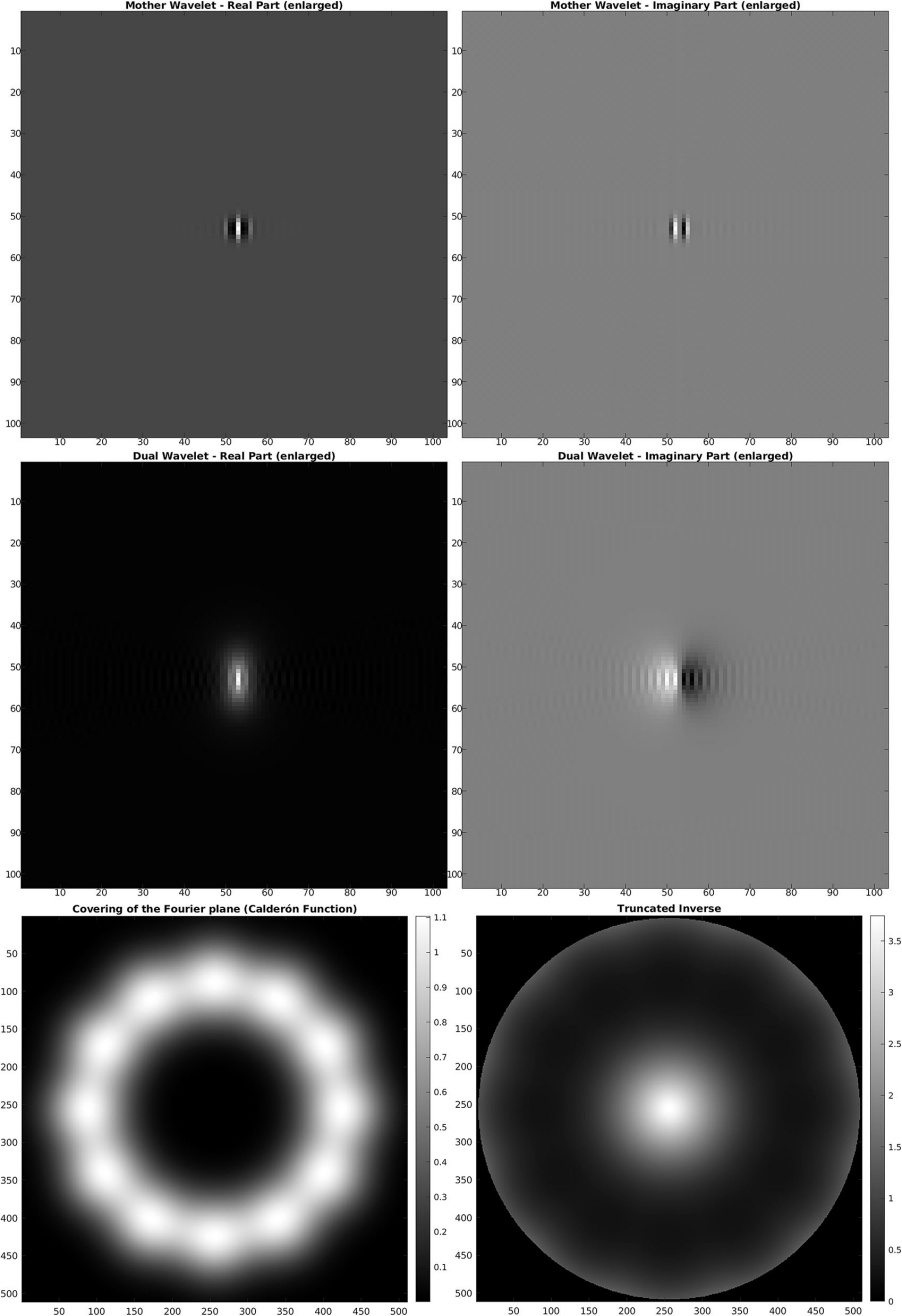
**Top:** mother wavelet ψ. **Center:** dual wavelet γ. **Bottom, left:** Calderón's function. **Bottom, right:** inverse of the Calderón's function in log_10_ scale, bandlimited with *R* = 252.

**Figure 3:**
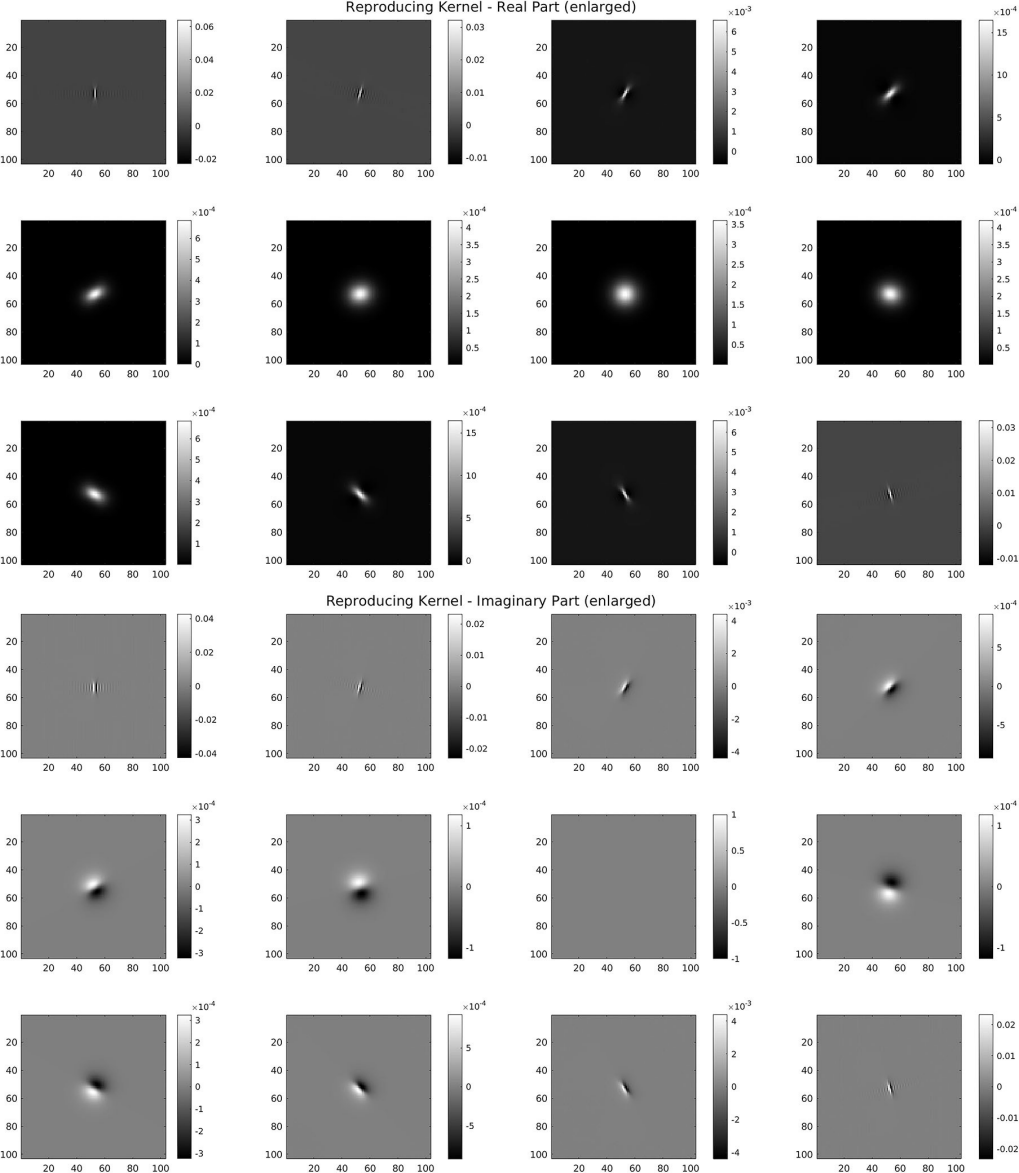
Reproducing kernel for the wavelet of Figure 2. **Top:** real part for the 12 angles. **Bottom:** imaginary part for the 12 angles.

For the discrete *SE*(2) transform, we have chosen a discretization of 𝕊^1^ with 12 angles, so that, with respect to the notation of the previous section, we have *N* = 512 and *M* = 12. We have also chosen the parameter values *s* = 51, *p* = 170, *R* = 252. The mother wavelet ψ and dual wavelet γ produced by these parameters are shown in Figure 2, top and center, on a crop of the full 512 × 512 space for better visualization. We stress that the stability of the transform and the numerical accuracy of the projection (Equation 16) depend only on the behavior of the Calderón's function, while the accuracy of image representation under bandlimiting with radius *R* depends on the decay of the spectra of the images. The Calderón's function *C*_ψ_, computed as in Equation (15), is shown in Figure 2, bottom left: the chosen parameters define a ratio *B*/*A* ≈ 6 · 10^3^, corresponding to a condition number for *W*_ψ_ of less than 10^2^. In Figure 2, bottom right, we have shown in log_10_ scale the multiplier χ_*B*_*R*__/*C*_ψ_ that defines the dual wavelet γ as in Equation (11), and in particular, we can see the bandlimiting radius, to be compared with the spectra of Figure 1. For visualization purposes, in Figure 3, we have shown in spatial coordinates the integral kernel defining the projection (Equation 16), which is the reproducing kernel for the discrete *SE*(2) transform. Its real and imaginary parts are shown on the same crop used to display the wavelet of Figure 2.

**Figure 4:**
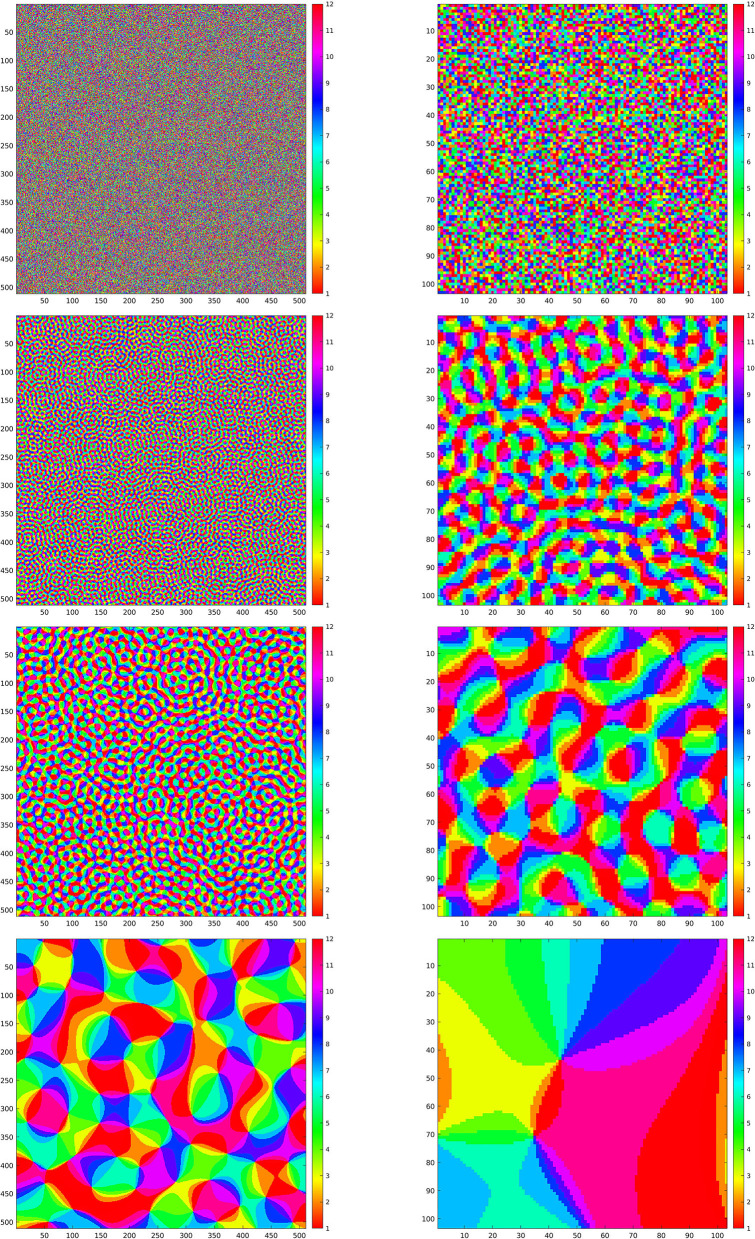
**Left column:** maps Θ for the simulations in Figures 5–8. **Right column:** enlargements of the same maps. First line: purely random Θ. Second, third, and fourth line: maps Θ_ρ_ generated according to Equation (23) with ρ respectively given by ρ = 0.8, ρ = 0.4, and ρ = 0.06.

We have implemented iteration (Equation 18) for the restriction of this discrete *SE*(2) transform to different types of feature maps Θ, shown in Figure 4. We will comment below each one of these cases. We have chosen to illustrate the effect of that iteration as follows. We have computed the sequence ${\left\{H_{n}\right\}}_{n=1}^{\nu }$ as in Equation (21), for a number ν of iterations, and applied the inverse *SE*(2) transform $W_{\gamma }^{*}$ to each *H*_*n*_. This allows us to obtain real images that are directly comparable with the original ones. We then have shown $W_{\gamma }^{*}H_{1}$, representing the first image that can be directly retrieved from the feature parameters, and $W_{\gamma }^{*}H_{\nu }$, that is the image obtained when we stopped the iteration. Moreover, as a measure of reconstruction error, we have considered the following rescaling of the Euclidean norm, at each step *n* ∈ {1, …, ν}:


(22) $$\begin{array}{l} \Delta_{n}=100*{\left(\frac{1}{N^{2}}\underset{x\in {\mathbb{Z}}_{N}\times {\mathbb{Z}}_{N}}{\sum }|f_{i}\left(x\right)-W_{\gamma }^{*}H_{n}\left[f_{i}\right]\left(x\right){|}^{2}\right)}^{\frac{1}{2}}/255\\ \;=100*\frac{\left||f_{i}-W_{\gamma }^{*}H_{n}\left[f_{i}\right]|\right|}{255*N}. \end{array}$$

This adimensional quantity measures a % error obtained as the average square difference by pixel of an image *f*_*i*_ in the dataset from its *n*-steps reconstruction $W_{\gamma }^{*}H_{n}\left[f_{i}\right]$, divided by the size of the admissible pixel range for 8 bit images, which is {0, …, 255}.

### 4.1. First Feature Map: Purely Random Selection of Angles

**Figure 5:**
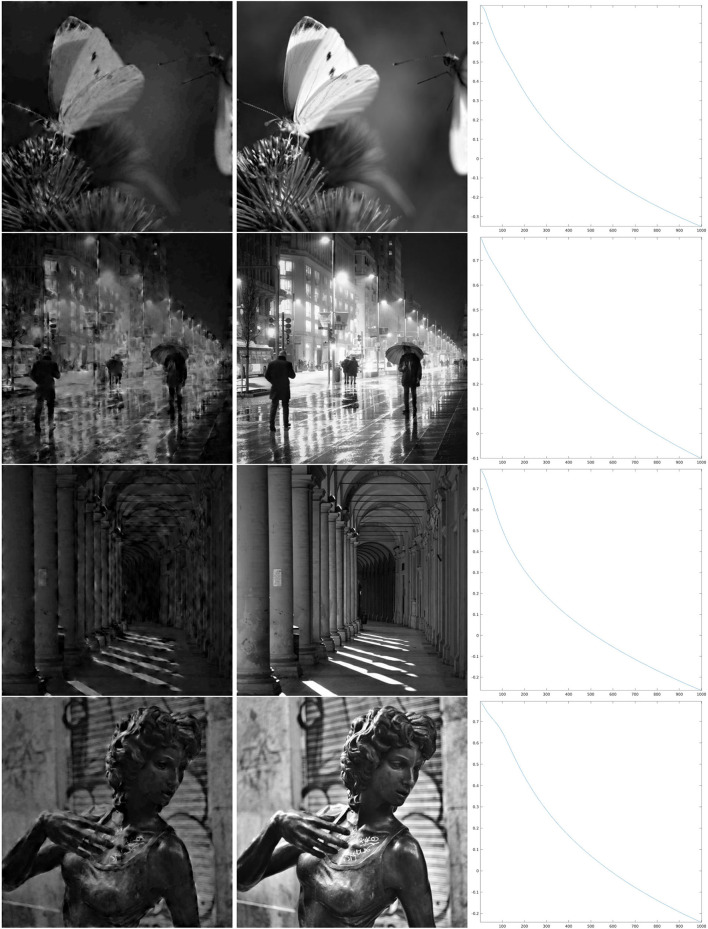
Iteration on the images 1–4 of Figure 1 for the purely random Θ shown in the first line of Figure 4. **Left:** The first step of the iteration. **Center:** after 1,000 iterations. **Right:** log_10_(Δ_*n*_), for the error (Equation 22).

The first map that we have considered is a Θ : ℤ_*N*_ × ℤ_*N*_ → ℤ_*M*_ that, for each *x* ∈ ℤ_*N*_ × ℤ_*N*_, simply chooses one value in ℤ_*M*_ as a uniformly distributed random variable. This map is shown in the first line of Figure 4, left, and in the first line of Figure 4, right, we have shown an enlargement to the same crop at which the wavelets in Figure 2 and the reproducing kernel in Figure 3 are shown. In Figure 5, we have shown the images resulting from $W_{\gamma }^{*}H_{1}$ and $W_{\gamma }^{*}H_{1000}$, and the evolution of the error (Equation 22) in log_10_ scale, respectively in the left, center, and right column, for the first four images of Figure 1. In this case, we can see that the error Δ_*n*_ goes beyond 1%, indicated by 0 on the *y*-axis, in just about 500 iterations. As a remark, feature maps that are similar to such configurations are commonly encountered in rodents (refer to e.g., Ho et al., 2021 and references therein).

### 4.2. Pinwheel-Shaped Feature Maps

Next, we present the results for three selection maps Θ that are pinwheel-shaped, as it is commonly observed for orientation and direction preference maps of V1 in primates and other mammals. These maps can be constructed as follows Petitot (2017): for ρ ∈ ℝ^+^, let ϕ_ρ_ : ℤ_*N*_ × ℤ_*N*_ → ℂ be given by


$$\phi_{\rho }\left(x\right)=\int_{0}^{2\pi }e^{i\left(\rho \left(x_{1}\cos\left(\alpha \right)+x_{2}\sin\left(\alpha \right)\right)+\Gamma \left(\alpha \right)\right)}d\alpha$$

where Γ is a purely random process with values in [0, 2π). The maps Θ_ρ_ : ℤ_*N*_ × ℤ_*N*_ → ℤ_*M*_ that we have considered are obtained by


(23) $$\begin{array}{l} \Theta_{\rho }\left(x\right)=\lfloor \frac{M}{2\pi }\text{angle}\left(\phi_{\rho }\left(x\right)\right)\rfloor \end{array}$$

where angle(*z*) is the phase of a complex number *z* ∈ ℂ, and ⌊*t*⌋ is the integer part of a real number *t*. The resulting maps are quasiperiodic, with a characteristic correlation length that corresponds to the fact that the spectrum of ϕ_ρ_ is concentrated on a ring of radius $\frac{\rho }{2\pi }$. The main feature of those maps Θ_ρ_ is that they possess points, called pinwheel points, around which all angles are mapped, and these points are spaced, on average, by a distance of 2π/ρ (refer to e.g., Petitot, 2017 and references therein).

In the second, third, and fourth line of Figure 4, left, we have shown the resulting maps Θ_ρ_ with ρ, respectively, given by ρ = 0.8, ρ = 0.4, and ρ = 0.06. On the right column, we have shown an enlargement to the same crop used before.

**Figure 6:**
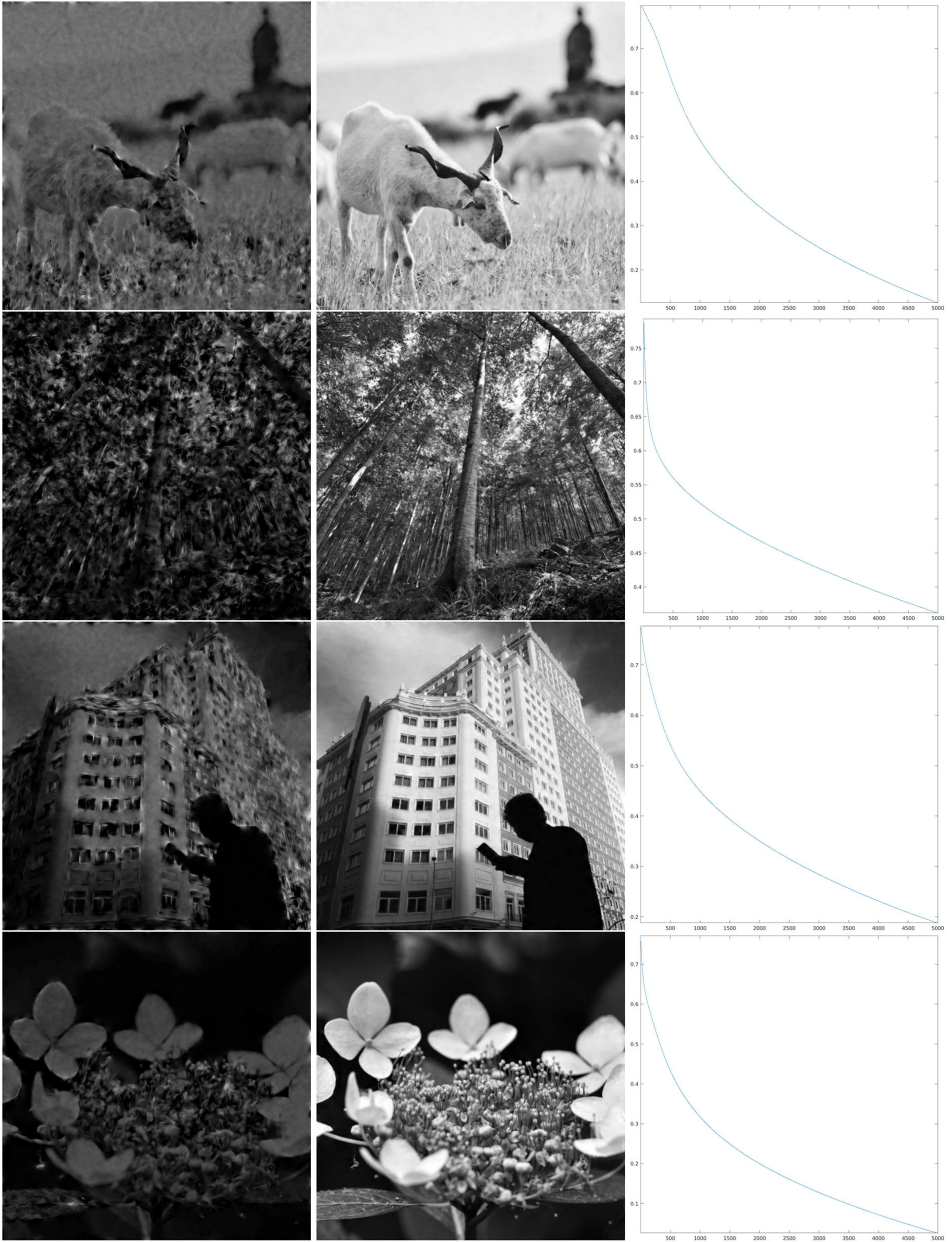
Iteration on the images 5–8 of Figure 1 for Θ_0.8_ shown in the second line of Figure 4. **Left:** The first step of the iteration. **Center:** after 5,000 iterations. **Right:** log_10_(Δ_*n*_), for the error (Equation 22).

**Figure 7:**
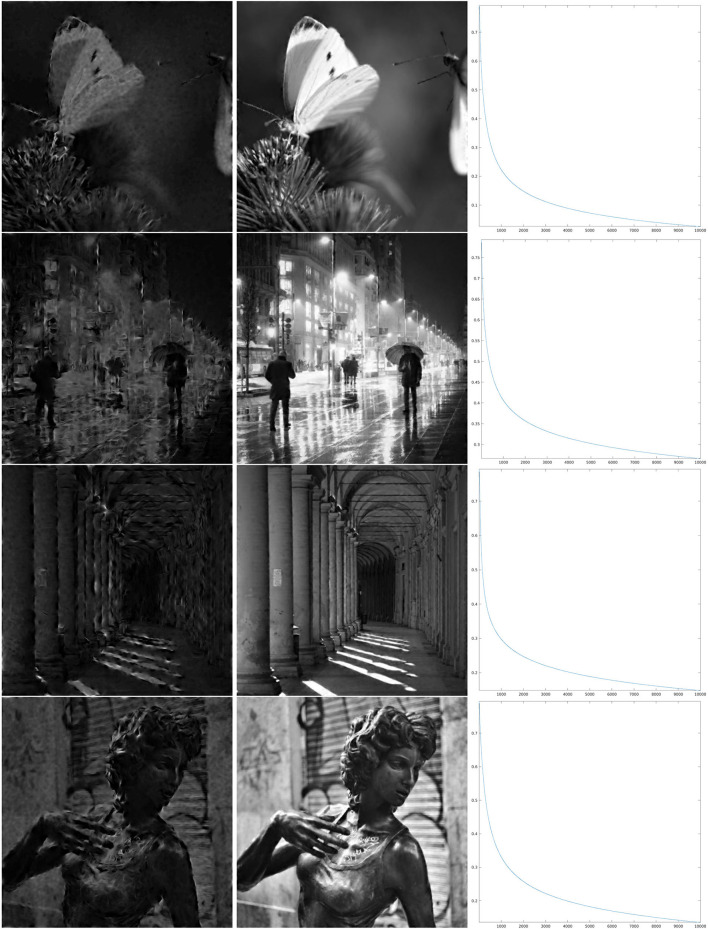
Iteration on the images 1–4 of Figure 1 for Θ_0.4_ shown in the third line of Figure 4. **Left:** The first step of the iteration. **Center:** after 10,000 iterations. **Right:** log_10_(Δ_*n*_), for the error (Equation 22).

**Figure 8:**
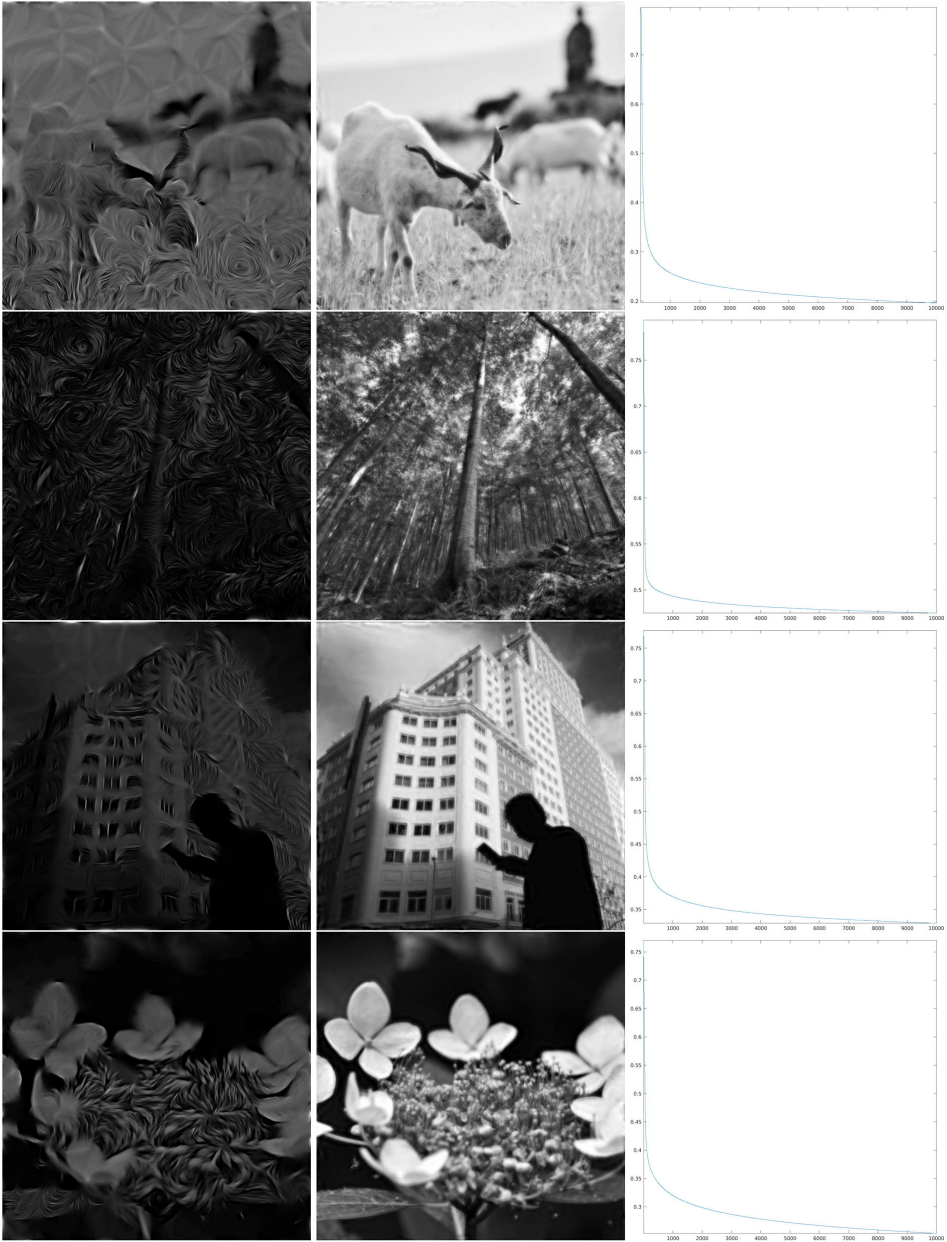
Iteration on the images 5–8 of Figure 1 for Θ_0.06_, shown in the fourth line of Figure 4. **Left:** The first step of the iteration. **Center:** after 10,000 iterations. **Right:** log_10_(Δ_*n*_), for the error (Equation 22).

The results of the iteration are presented, as described above, in Figures 6–**8**, whose structure is the same as in Figure 5 with the exception of the number of iterations, which is now larger.

In order to discuss these results, we first recall that the correlation length of orientation preference maps has been often related to the *size* of receptive fields, as a “coverage” constraint to obtain a faithful representation of the visual stimulus (refer to Swindale, 1991; Swindale et al., 2000; Bosking et al., 2002; Keil and Wolf, 2011; Barbieri et al., 2014 and references therein). However, neither mathematical proof of this principle in terms of image reconstruction has been given so far, nor has the word *size* received a more quantitative meaning within the models of type (Equation 1), and we have not tried to give any more specific meaning either. However, for the three cases that we present, by comparing the crops of the maps Θ_ρ_ in Figure 4 with the wavelet ψ in Figure 2, we can see that for ρ = 0.8, the correlation length of the map Θ_ρ_ is approximately similar to what we could call effective support of the receptive field, while for ρ = 0.4, we have that the area of influence of the receptive field does not include two different pinwheel points, and for ρ = 0.06, the two scales are very different. Heuristically, one could then be led to think that the reconstruction properties in the three cases may present qualitative differences. For example, that condition (Equation 14), or its discrete counterpart (Equation 20), may hold in the first case and may not hold in the third case.

As can be seen from the numerical results of the proposed algorithm, there is actually a difference in the behavior of the decay. For larger values of the parameter ρ, when the map Θ_ρ_ is more similar to the purely random selection described above, the decay of the error is faster, while for smaller values of ρ the decay is slower, but nevertheless, the error appears to be monotonically decreasing. In the presented cases, for ρ = 0.8, we can see in the right column of Figure 6 that in about 2,000 iterations the error decay appears to enter an exponential regime, which is rectilinear in the log_10_ scale. We see in Figure 7 that it takes roughly twice as many iterations for ρ = 0.4 to enter the same regime. On the other hand, for ρ = 0.06, we can see in Figure 8 that after a relatively small number of iterations the decay becomes very small and does not seem to become exponential even after 10,000 iterations. However, visual inspection of the results (which “measures” the error in a different way than the Euclidean norm) in this last case, show that the starting image appears to be qualitatively highly corrupted, while the image obtained after the iteration was stopped is remarkably true to the original one and does not display evident artifacts away from the boundaries.

### 4.3. Selection of a Single Orientation: Deconvolution

**Figure 9:**
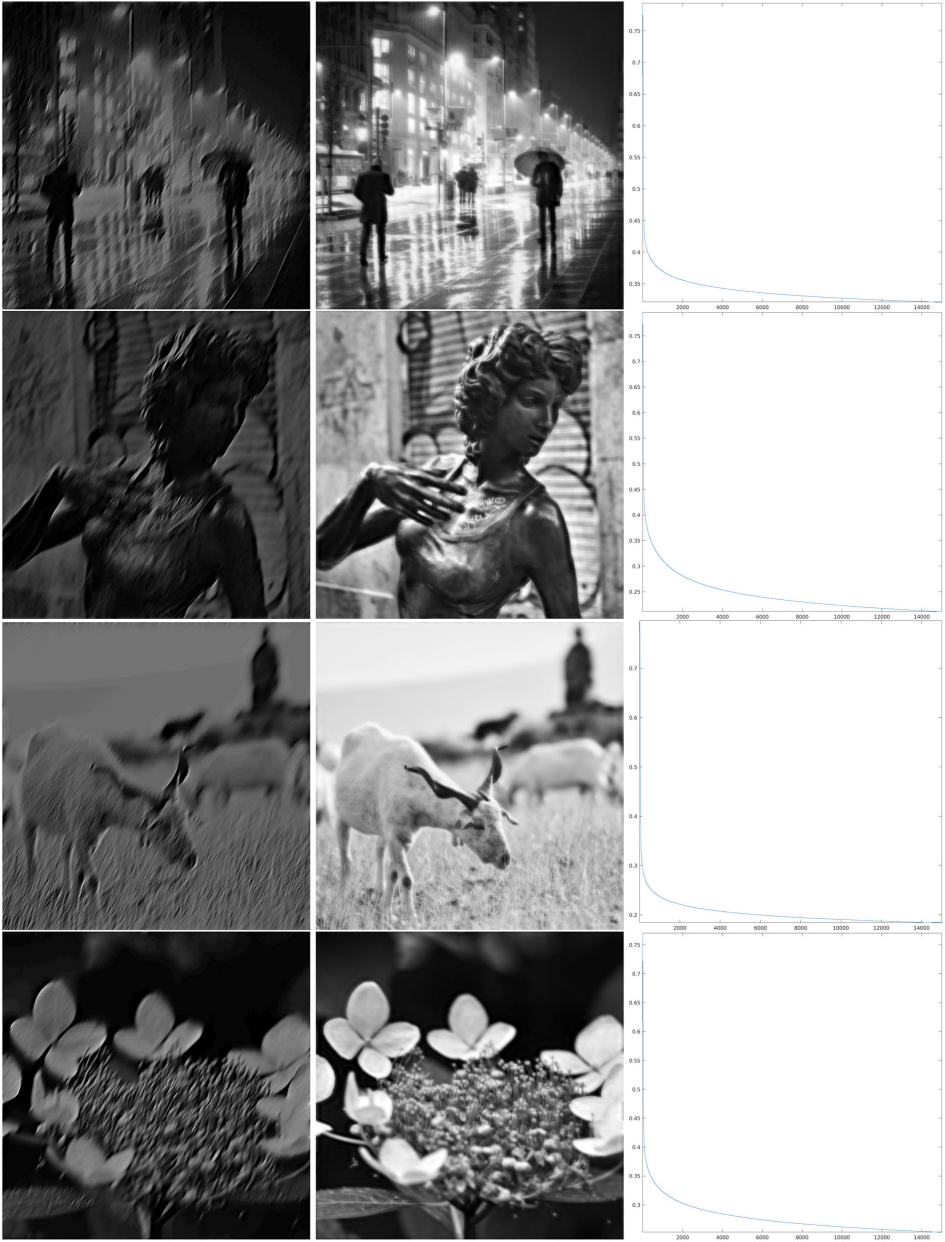
Iteration for the *SE*(2) deconvolution by ψ on images of Figure 1. **Left:** The first step of the iteration. **Center:** after 15,000 iterations. **Right:** log_10_(Δ_*n*_), for the error (Equation 22).

The surprisingly good performance in the reconstruction problem for the last map Θ_0.06_ has motivated a performance test for an additional feature selection map, given by a function Θ that is independent of *x*, hence selecting just one angle in the *SE*(2) transform. In the same fashion as seeing the purely random distribution as a limiting case for large ρ of the pinwheel-shaped maps, this can be considered as a limiting case for small ρ. However, keeping only the values of *W*_ψ_*f* for a single angle θ concretely corresponds to performing a convolution with one function ψ_θ_, and aiming to reconstruct *f* is actually a deconvolution problem. In this case, the frequency behavior of the convolution filter is that of a Gaussian centered away from the origin, and its shape can be observed in the Calderón's function of Figure 2, which clearly shows the sum of 12 such Gaussians. We have shown the reconstruction results for this problem, using now 15,000 iterations, in Figure 9. The error decay seems to share, qualitatively, much of the same properties of the case ρ = 0.06.

## 5. Conclusions

In this article, we have proposed an elementary iterative technique to address the problem of the reconstruction of images from a fixed reduced set of values of its *SE*(2) group wavelet transform. We have formally defined these restrictions in terms of cortical maps, as this problem is inspired by visual perception, since the *SE*(2) symmetry and the associated integral transform have proved to be relevant for mathematical modeling of V1, refer to e.g., (Petitot and Tondut, 1999; Paul C. Bressloff, 2002; John Zweck, 2004; Citti and Sarti, 2006, 2015). Moreover, the presented numerical simulations directly compare with the studies on the relationship between cortical maps and the efficiency of single cell encoding of information in terms of coverage (Swindale, 1991; Swindale et al., 2000; Bosking et al., 2002; Keil and Wolf, 2011; Barbieri et al., 2014). Indeed, as a result of the main theorem, and for cortical maps as formally defined in this article, we can see from the proposed implementation that, when a pinwheel structure is chosen, the relationship between the average distance between pinwheel centers and the size of receptive fields influences the invertibility of the restricted *SE*(2) transform.

A possible interpretation of the proposed iteration with a kernel defined by the *SE*(2) group as a neural computation in V1 comes from the modeling of the neural connectivity as a kernel operation (Wilson and Cowan, 1972; Ermentrout and Cowan, 1980; Citti and Sarti, 2015; Montobbio et al., 2018), especially if considered in the framework of a neural system that aims to learn group invariant representations of visual stimuli (Anselmi and Poggio, 2014; Anselmi et al., 2020). A direct comparison of the proposed technique with kernel techniques recently introduced with radically different purposes in Montobbio et al. (2018) and Montobbio et al. (2019) shows, however, two main differences at the level of the kernel that is used: here, we need the dual wavelet to build the projection kernel, and the iteration kernel effectively contains the feature maps. On the other hand, a possible application is the inclusion of a solvability condition such as Equation (14) as iterative steps within learning frameworks such as those of Anselmi et al. (2019) and Anselmi et al. (2020). From the point of view of actual neural implementation, even if it was possible to see a formal analogy between the proposed algorithm and a neural field equation, we believe that more has to be done concerning the performances and that the proposed mechanism has to be considered as a much more elementary procedure than the ones that could take place in a real visual cortex. For example, while the proposed iteration is faster than typical convex optimization implementations used for other completion problems, it still requires a large number of iterations even in the exponential regime. Improvements could be searched in several directions, e.g., by including non-classical behaviors in neural modeling.

We would like to observe also that, since the proof of convergence of this technique is general, it could be applied to other problems with a similar structure. The computational cost essentially relies on the availability of efficient methods to implement the two projections that define the problem in the discrete setting, as it happens to be the case for the setting studied in this article. In particular, similar arguments could be applied to other wavelet transforms based on semidirect product groups ℝ^*d*^ ⋊ *G*, with *G* a subgroup of *GL*_*d*_(ℝ) that defines what is sometimes referred to as local features, and to sampling projections obtained for example, but not only, from other types of feature maps Θ : ℝ^*d*^ → *G*. From the dimensional point of view, in the discrete setting the selection of local features with a feature map can be seen as downsampling that allows one to maintain in the transformed space the same dimension of the input vector. This is often a desirable property and it is already commonly realized e.g., by the MRA decomposition algorithm of classical wavelets or by the pooling operation in neural networks. Moreover, its apparent stability of convergence seems to suggest that this operation can be performed a priori, without the need for a previous study of the solvability of the problem.

Several questions remain open after this study. Probably the most fundamental one is the characterization of those maps Θ that, for a given mother wavelet ψ, satisfy the solvability condition (Equation 14). In terms of the study of the convergence of the project and replace iteration, it is plausible that one could obtain convergence under weaker conditions than Equation (20), even if maybe to a different solution, as it appears to happen in some of the numerical simulations presented.

## Data Availability Statement

The original contributions presented in the study are included in the article/supplementary material, further inquiries can be directed to the corresponding author.

## Author Contributions

The author confirms being the sole contributor of this work and has approved it for publication.

## Funding

This project has received funding from the European Union's Horizon 2020 Research and Innovation Programme under the Marie Skłodowska-Curie grant agreement No 777822, and from Grant PID2019-105599GB-I00, Ministerio de Ciencia e Innovación, Spain.

## Conflict of Interest

The author declares that the research was conducted in the absence of any commercial or financial relationships that could be construed as a potential conflict of interest.

## Publisher's Note

All claims expressed in this article are solely those of the authors and do not necessarily represent those of their affiliated organizations, or those of the publisher, the editors and the reviewers. Any product that may be evaluated in this article, or claim that may be made by its manufacturer, is not guaranteed or endorsed by the publisher.

## Appendix: Proofs of the Main Statements

This section contains the proofs of the formal statements introduced in the text.

**S****ketch of the proof of theorem** **2**. By Fourier Convolution Theorem, Plancherel's theorem, and the definition of $H_{R}$, we have

$$\begin{array}{l} \int_{{𝕊}^{1}}\int_{{\mathbb{R}}^{2}}|W_{\text{Ψ}}f\left(x,\theta \right){|}^{2}dxd\theta =\int_{{𝕊}^{1}}\int_{{\mathbb{R}}^{2}}|\hat{\psi_{\theta }}\left(\xi \right){|}^{2}|\hat{f}\left(\xi \right){|}^{2}d\xi d\theta \\ \;=\int_{B_{R}}\left(\int_{{𝕊}^{1}}|\hat{\psi_{\theta }}\left(\xi \right){|}^{2}d\theta \right)|\hat{f}\left(\xi \right){|}^{2}d\xi . \end{array}$$

Thus, (9) is equivalent to the so-called frame condition

(A1)
$$\begin{array}{l} A{∥f∥}_{L^{2}\left({\mathbb{R}}^{2}\right)}^{2}\leq {∥W_{\psi }f∥}_{L^{2}\left({\mathbb{R}}^{2}\times {𝕊}^{1}\right)}^{2}\leq B{∥f∥}_{L^{2}\left({\mathbb{R}}^{2}\right)}^{2} \end{array}$$

for all $f\in H_{R}$, which is equivalent [refer to e.g., Ali et al. (1993)] to *W*_Ψ_ being bounded and invertible on $H_{R}$.     □

**S****ketch of the** **P****roof of** **T****heorem** **3**. Observe first that $\hat{\gamma_{\theta }^{†}}=\bar{\hat{\gamma_{\theta }}}=\frac{\chi_{B_{R}}\left(\xi \right)}{C_{\psi }\left(\xi \right)}\bar{\hat{\psi_{\theta }}\left(\xi \right)}.$ Thus, recalling (7) and using Fourier Convolution Theorem, we can compute

$$\begin{array}{l} \hat{W_{\gamma }^{*}W_{\psi }f}\left(\xi \right)=\int_{{𝕊}^{1}}\hat{f}\left(\xi \right)\frac{\chi_{B_{R}}\left(\xi \right)}{C_{\psi }\left(\xi \right)}|\hat{\psi_{\theta }}\left(\xi \right){|}^{2}d\theta =\hat{W_{\psi }^{*}W_{\gamma }f}\left(\xi \right)\\ \;=\hat{f}\left(\xi \right)\chi_{B_{R}}\left(\xi \right) \end{array}$$

which proves (i). To prove (ii), one needs to show that the elements of $W_{\psi }\left(H_{R}\right)$ are continuous functions, that $W_{\psi }\left(H_{R}\right)$ $W_{\psi }W_{\gamma }^{*}$ is self adjoint and idempotent, and that (12) holds. The continuity can be obtained as a consequence of the continuity of the unitary representation (6) and, by the same arguments used to prove (i) we can easily see that $W_{\gamma }W_{\psi }^{*}=W_{\psi }W_{\gamma }^{*}$. On the other hand, (i) implies that $W_{\psi }W_{\gamma }^{*}W_{\psi }f=W_{\psi }f$ for all $f\in H_{R}$, hence $W_{\psi }W_{\gamma }^{*}F=F$ for all $F\in W_{\psi }\left(H_{R}\right)$. Equation (12) can be obtained directly by (7) and the definition of *W*_ψ_.     □

**P****roof of** **L****emma** **4**. The system (19) is of course solved by $F=\tilde{F}$, so we only need to prove that, for all $\tilde{F}\in \text{Ran}\left(P\right)$, this solution is unique if and only if (20) holds. In order to see this, let 𝕊 ∈ *V* be a solution to (19), and denote by $E=\tilde{F}-S$. Then *E* ∈ Ran(*P*) ∩ Ker(*Q*). Hence, (20) holds if and only if *E* = 0, i.e., the only solution to (19) is $F=\tilde{F}$.     □

**P****roof of** **T****heorem** **5**. Denoting by *H*_1_ = *PF*_0_, we can rewrite the iteration (21) as two separate iterations, generating the two sequences {_*F*_*n*_}*n* ∈ ℕ_, {_*H*_*n*_}*n* ∈ ℕ_ as follows:

(A2)
$$\begin{array}{l} \{\begin{array}{l} H_{n+1}=PQ^{⊥}H_{n}+H_{1}=\ldots =\overset{n}{\underset{k=0}{\sum }}{\left(PQ^{⊥}\right)}^{k}H_{1}\\ \;F_{n}=Q^{⊥}PF_{n-1}+F_{0}=\ldots =\overset{n}{\underset{k=0}{\sum }}{\left(Q^{⊥}P\right)}^{k}F_{0}. \end{array} \end{array}$$

If (20) holds, then ||*PQ*^⊥^|| < 1 and ||*Q*^⊥^*P*|| < 1, because $\text{Ran}\left(\mathbb{P}\right)\cap \text{Ran}\left({O_{\Theta }}^{⊥}\right)=\text{Ran}\left(\mathbb{P}\right)\cap \text{Ker}\left(O_{\Theta }\right)=\left\{0\right\}$. Hence, the existence of the limits $H=\underset{n\to \infty }{\lim}H_{n}$ and $F=\underset{n\to \infty }{\lim}F_{n}$ is due to the convergence of the Neumann series [refer to e.g., Riesz and Sz.-Nagy (1990)]. Thus, getting rid of the notations $F_{0}=Q\tilde{F}$ and *H*_1_ = *PF*_0_, we have that the limits *H* and *F* are the solutions to the equations

$$\begin{array}{l} \{\begin{array}{c} \left(1-PQ^{⊥}\right)H=PQ\tilde{F}\\ \left(1-Q^{⊥}P\right)F=Q\tilde{F}. \end{array} \end{array}$$

We can now see that $F=\tilde{F}$, because this equation reads

$$\begin{array}{l} {\left(1-Q^{⊥}P\right)}^{-1}Q\tilde{F}=\tilde{F} \end{array}$$

and, using that $\tilde{F}\in \text{Ran}\left(P\right)$, we can rewrite it as

$$\begin{array}{l} Q\tilde{F}=\left(1-Q^{⊥}P\right)\tilde{F}=\tilde{F}-Q^{⊥}\tilde{F} \end{array}$$

which is true by definition of *Q*^⊥^ = **1** − *Q*. Moreover, by taking the limit in the first equation of (21), and using again that $\tilde{F}\in \text{Ran}\left(P\right)$, we obtain

$$\begin{array}{l} H=PF=P\tilde{F}=\tilde{F}. \end{array}$$

Finally, the convergence of (18) is exponential because, using the series expression (A2) for $\tilde{F}=F$,

$$\begin{array}{l} ∥\tilde{F}-F_{n}∥=∥\overset{\infty }{\underset{k=0}{\sum }}{\left(Q^{⊥}P\right)}^{k}F_{0}-\overset{n}{\underset{k=0}{\sum }}{\left(Q^{⊥}P\right)}^{k}F_{0}∥\\ \;=∥\overset{\infty }{\underset{k=n+1}{\sum }}{\left(Q^{⊥}P\right)}^{k}F_{0}∥=∥{\left(Q^{⊥}P\right)}^{n+1}\overset{\infty }{\underset{k=0}{\sum }}{\left(Q^{⊥}P\right)}^{k}F_{0}∥\\ \;=∥{\left(Q^{⊥}P\right)}^{n+1}F∥\leq ∥Q^{⊥}P{∥}^{n+1}∥F∥ \end{array}$$

A similar argument applies to $\left||\tilde{F}-H_{n}|\right|$.     □
